# Compensatory Role of Inositol 5-Phosphatase INPP5B to OCRL in Primary Cilia Formation in Oculocerebrorenal Syndrome of Lowe

**DOI:** 10.1371/journal.pone.0066727

**Published:** 2013-06-21

**Authors:** Na Luo, Akhilesh Kumar, Michael Conwell, Robert N. Weinreb, Ryan Anderson, Yang Sun

**Affiliations:** 1 Glick Eye Institute, Department of Ophthalmology, Department of Biochemistry and Cell Biology, Department of Dermatology, Indiana University Indianapolis, Indiana, United States of America; 2 Department of Ophthalmology, University of California San Diego, San Diego, California, United States of America; 3 Department of Pediatrics, Indiana University Indianapolis, Indiana,United States of America; Institute of Molecular and Cell Biology, Singapore

## Abstract

Inositol phosphatases are important regulators of cell signaling, polarity, and vesicular trafficking. Mutations in *OCRL*, an inositol polyphosphate 5-phosphatase, result in Oculocerebrorenal syndrome of Lowe, an X-linked recessive disorder that presents with congenital cataracts, glaucoma, renal dysfunction and mental retardation. *INPP5B* is a paralog of *OCRL* and shares similar structural domains. The roles of *OCRL* and *INPP5B* in the development of cataracts and glaucoma are not understood. Using ocular tissues, this study finds low levels of INPP5B present in human trabecular meshwork but high levels in murine trabecular meshwork. In contrast, OCRL is localized in the trabecular meshwork and Schlemm’s canal endothelial cells in both human and murine eyes. In cultured human retinal pigmented epithelial cells, INPP5B was observed in the primary cilia. A functional role for INPP5B is revealed by defects in cilia formation in cells with silenced expression of *INPP5B*. This is further supported by the defective cilia formation in zebrafish Kupffer’s vesicles and in cilia-dependent melanosome transport assays in *inpp5b* morphants. Taken together, this study indicates that *OCRL* and *INPP5B* are differentially expressed in the human and murine eyes, and play compensatory roles in cilia development.

## Introduction

Oculocerebrorenal syndrome of Lowe (MIM #309000) is an X-linked recessive disorder characterized by the presence of congenital cataracts, glaucoma, mental retardation, and renal dysfunction [1], [2], [3]. Lowe syndrome results from mutations in *OCRL*, a type II inositol polyphosphate 5-phosphatase [2]. Patients with Lowe syndrome typically present with bilateral congenital discoid cataracts, which may be caused by disorganization of embryonic lens epithelium [4], [5]. Glaucoma is present in approximately 47% (44 of 93) of Lowe syndrome patients [4]. When observed at birth, glaucoma development in the Lowe syndrome patients represent a form of trabeculodysgenesis, which is the abnormal development of the trabecular meshwork that regulates aqueous humor outflow from the eye [4]. Produced by the nonpigmented layer of epithelial cells (NPCE) in the ciliary body, the major route of aqueous humor drainage is via the trabecular meshwork and then into the Schlemm’s canal, a monolayer of endothelial cells, where it finally drains out of the eye by joining the venous blood vessels [6]. Over 208 mutations in *OCRL* have been described with a wide range of phenotypes that involve multiple organ systems [7]. However, very little is known how defects in *OCRL* results in cellular dysfunction that underlies cataracts formation and the defective flow of aqueous humor that leads to congenital glaucoma.

Type II inositol polyphosphate 5-phosphatase (*INPP5B*), a paralog of *OCRL*, is also an important regulator of intracellular phosphoinositide levels [8]. INPP5B and OCRL share 45% sequence identity, similar domain architecture, and substrate specificity [9], [10], [11], [12]. Both INPP5B and OCRL are recruited to the membrane of endosomes and plasma membrane, where OCRL has been shown to be important in clathrin-mediated trafficking [9], [13], [14], [15], [16], [17], [18]. Although *Ocrl* knockout mice do not display the typical symptoms of Lowe syndrome, *Inpp5b* knockout mice exhibit male sterility, which has limited functional studies to distinguish their roles in Lowes syndrome [19], [20], [21], [22], [23]. The embryonic lethality of *Ocrl^−/−^*:*Inpp5b^−/−^* double knockout mice suggests that these genes have overlapping functions [19].

Phosphoinositides and the phosphatases that regulate their metabolism have key roles in the development of the primary cilia [24], [25], [26], [27], [28], [29]. A subcellular organelle present in almost all post-mitotic cells, the primary cilium is formed by a microtubule-based axoneme and a basal body, which is the nucleating center for the axoneme [30], [31]. Disruptions in the primary cilium can result in a range of clinical abnormalities that collectively are known as ciliopathies [32], [33]. A Type IV inositol polyphosphate 5-phosphatase, INPP5E, has been localized to the cilium and mutations in *INPP5E* have been found in Joubert syndrome, which presents with retinitis pigmentosa, kidney cysts, and mental developmental delays [26], [27], [34]. Recently several groups have found OCRL in the primary cilia, supporting the role of inositol 5-phosphatases in cilia formation [35], [36], [37].

This study analyzes the intracellular localization and interactions of OCRL and INPP5B in ocular tissues responsible for cataract and glaucoma development. Based on genetic studies, the differential expression of *OCRL* and *INPP5B* are proposed to underlie disease pathogenesis of the ocular phenotypes. Further, given the roles of OCRL and INPP5E in the primary cilium, INPP5B was investigated for roles in the primary cilium. INPP5B indeed localizes to the cilia but unlike OCRL, this requires an intact C-terminal CAAX prenylation domain. Knockdown of *inpp5b* results in zebrafish morphants with reduced cilia length in Kupffer’s vesicle (KV) and other cilia-related phenotypes. Together, INPP5B is found to have an overlapping function with OCRL in cilia development.

## Methods and Materials

### Reagents

Anti-OCRL antibodies have been previously described [35]. Anti-INPP5B antibody was purchased from Proteintech (Immunogen: 1–309 amino acid of human INPP5B)(Chicago, IL). Antibodies against gamma-tubulin, and acetylated alpha-tubulin were purchased from Sigma (St. Louis, MO). Secondary antibodies AlexaFluor 488 and 546 -conjugated donkey anti-mouse IgG (1∶1000), Cy3-conjugated donkey anti-mouse IgG (1∶500), horseradish peroxidase-conjugated goat anti-rabbit and anti-mouse IgG were obtained from Jackson ImmunoResearch Laboratories, Inc (West Grove, PA). IRDye goat anti-mouse and anti-rabbit (680 and 800) were obtained from Li-cor Bioscience (Lincoln, NB).

### DNA Constructs

*FLAG-INPP5B*, *FLAG-Inpp5b* and lentiviral *GFP-Inpp5b* were generated using Creator (Clontech) based vectors as described [38]. *FLAG-INPP5B*-*delta*-*CAAX*, *FLAG-Inpp5b*-*delta*-*CAAX* and lentiviral *GFP-Inpp5b*-*delta*-*CAAX* mutant were generated using QuikChangeII kit from Stratagene (Santa Clara, CA).

### Human Ocular Specimens and Animal Tissue

All experiments were conducted in accordance with the Association for Research in Vision and Ophthalmology (ARVO) Statement on the Use of Animals in Ophthalmic and Vision Research. All procedures in rats were approved by the Institutional Animal Care and Use Committees at Indiana University (Study number #0000003163). Zebrafish studies were approved by the Institutional Animal Care and Use Committees at Indiana University (Study number #3843). Human eyes specimens were obtained with approval of Health Sciences and Behavioral Sciences Institutional Review Boards (IRB-HSBS) of University of Michigan (HUM00034652) and was previously published [35]. Mice *mOcrl^−/−^:mInpp5b^−/−^:hINPP5B* ^+/+^ (ocular samples were generous gifts of Dr. R. Nussbaum, University of California, SF, CA) were previously described [21].

### Cell Culture and Transfection

hTERT-RPE1 (American Type Culture Collection) were grown in DMEM/F-12+GlutaMAX (GIBCO) with 10% FCS, penicillin–streptomycin at 37 C in 5% CO_2_. Primary human trabecular meshwork (HTM) cells were generous gifts of Dr. Juanita Dortch-Carnes (Morehouse school of Medicine, Atlanta, GA) [39]. Normal human fibroblast (NHF) is a gift of Dr. Dan Spandau (Indiana University, Indianapolis, IN) as previously described [35]. Lowe 1676 and Lowe 3265 patient fibroblasts (Coriell Institute) have markedly decreased OCRL protein levels and were previously characterized [35]. Transfections were performed using Polyethylenimine according to previous published protocol [40].

### Immunohistochemistry and Immunofluorescence

Immunohistochemistry was performed by obtaining ten micrometer sections from formalin fixed paraffin-embedded tissue, stained with H&E as previously described [35]. Immunofluorescence slides were treated with paraformaldehyde (PFA) for fixation for 10 minutes at room temperature (RT) followed by permeabilization with 0.5% Triton X-100, then blocked with Phosphate buffered saline (PBS)/0.5% Bovine Serum Albumin (BSA)/10% Normal Goat Serum (NGS) for 30 minutes at RT. Cover slips were incubated with the primary antibodies overnight at 4°C, secondary antibodies for 45 minutes at RT, and mounted in ProLong Antifade reagent (Invitrogen). The hTERT-RPE1 cells were synchronized by serum starvation according to previous protocols [41]. To evaluate cilia length, cells were grown to confluence for 48 hours followed by serum deprivation, then fixed with 4% PFA and cilia growth was analyzed by immunostaining with acetylated α-tubulin from z-stacks (0.56 micron step size) taken with LEICA SP6 confocal microscope or Zeiss LSM-700 confocal microscope. Ciliary length was measured with NIH Image J software and statistical analysis was performed with SAS.

### Immunoblot Analysis

Cell lysates were subjected to SDS-PAGE on 10%–12% gel and transferred to nitrocellulose membrane (BioRad). Membranes were blocked in 5% non-fat dried milk in PBS. Primary and secondary antibodies were diluted in concentrations as described above [35]. Odyssey Imaging system (Li-Cor Bioscience) was used to analyze the immunoblots.

### Knockdown of INPP5B in Cells

INPP5B and control knockdown shRNA lentivirus was obtained from Santa Cruz Biotechnology (Santa Cruz, CA). Lentiviral particles were transduced into hTERT-RPE1 cells using standard protocols. Knockdown of INPP5B was verified by immunoblotting as described [42].

### Zebrafish Immunohistochemistry and KV Cilia Measurements

Zebrafish (wildtype strain: AB *Tubingen*) were raised and maintained at the Laboratory Animal Resource Center of Indiana University. All animal procedures were performed in accordance with the recommendations in the Guide for the Care and Use of Laboratory Animals of the NIH. The protocol was approved by the Indiana University Animal Care and Use committee (number 3843). All surgery was performed under paracaine anesthesia. Embryos were fixed overnight at 4°C in 4% paraformaldehyde and 1% sucrose in PBS. Embryos were dechorionated and washed with PBST for 6–8 times 10 min each. After blocking them for 2–4 hr with 10% NGS and 0.5% BSA, immunostaining was performed with 1∶200 anti-acetylated tubulin monoclonal antibody and 1∶500 AlexaFluor 546 donkey anti-mouse conjugate separately at 4°C overnight. KV cilia measurements were performed as described [43]. Cresyl violet staining were performed as described [42].

### Morpholino Antisense Oligonucleotides Knockdown and mRNA Rescue in Zebrafish

Antisense MOs were designed and purchased from Gene Tools, Inc. (Philo, OR). Two morpholinos were generated; *inpp5b* translational blocking MO (*inpp5b* MO: sequence CTCCTGAAACCCGCCATCAGCGTTC), mismatch (control MO: sequence ATGCGAAATCAAGGTTCGATCATCA), and *p53* (*p53* MO: sequence GCGCCATTGCTTTGCAAGAATTG) morpholino served as a negative control. Morpholinos stocks were dissolved at 1 mM in water: 2 or 4 nl of injection solution (0.25% phenol red) containing 125–500 mM morpholino was injected into fertilized eggs at the one- to two-cell stage using a pressure injector (Pressure System IIe, Toohey Company, Fairfield, NJ). Synthetic mRNA was prepared from linearized human DNA with Ambion mMessage mMachine high-yield Capped RNA transcription kit, purified with phenol-chloroform, and mRNA was coinjected for rescue experiments as previously described [35].

### Retrograde Melanosome Transport Assay

Melanosome transport assay was performed as described [42]. Briefly, zebrafish 5 dpf larvae were exposed to epinephrine (50 mg/ml, Sigma) at the final concentration of 2 mg/ml in a dark room, and melanosome retractions were observed under the brightfield microscope Leica DFC310 FX. The end of melanosome transport was marked when all melanosomes in the head were perinuclear.

## Results

### Localization of Inositol Phosphates to the Cilia in Ocular Tissues

**Figure 1:**
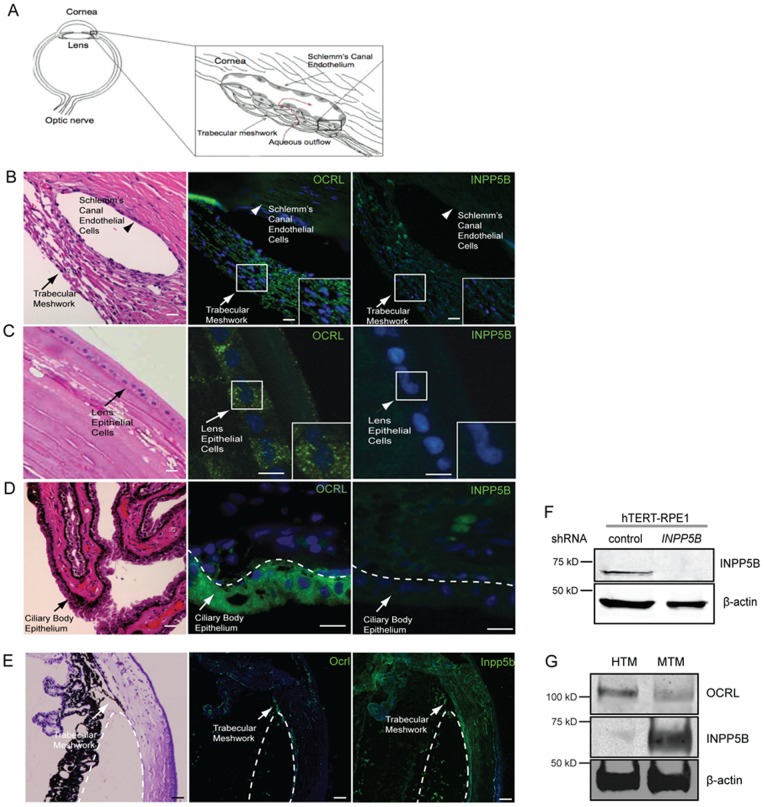
Localization of OCRL and INPP5B in human ocular tissue. (A) Diagram of trabecular meshwork and Schlemm’s canal endothelial cells (aqueous flow, red; trabecular meshwork, arrow; Schlemm’s canal endothelial cells, arrowhead). (B) Human eye sectioned and stained by H&E or immunofluorescence with anti-OCRL (green) or anti-INPP5B antibody (green), and DAPI (blue). Staining of trabecular meshwork cells in vesicular pattern (insert, arrow) and Schlemm’s canal (arrowhead). Scale bar 10 micron. (C) Lens epithelial cells (arrow) stained with H&E, anti-OCRL antibody (green) or anti-INPP5B antibody (green), and DAPI (blue). Scale bar 10 micron. (D) Ciliary body epithelial (arrowhead) cells stained with H&E, anti-OCRL antibody (green) or anti-INPP5B antibody (green), and DAPI (blue). Scale bar 10 micron. (E) Mouse eye sectioned and stained with H&E, anti-OCRL antibody (green) or anti-INPP5B antibody (green), and DAPI (blue). Staining of trabecular meshwork cells in vesicular pattern (dash line indicates border of trabecular meshwork). Scale bar 10 micron. (F) Immunoblot of *INPP5B* expression in 30 microgram lysates of hTERT-RPE1 shRNA knockdown cells compared to beta-actin. (G) Immunoblot analysis of 40 microgram lysates of human and mouse trabecular meshwork with anti-OCRL, anti-INPP5B and anti-beta-actin antibodies.

*OCRL* mutations have been found in Lowe syndrome, which usually presents with bilateral congenital cataracts and glaucoma [4]; however, the pathogenesis of these ocular phenotypes is not known. In the anterior segment of the eye, the trabecular meshwork and Schlemm’s canal endothelial cells regulate aqueous outflow and are often affected in glaucoma patients (Fig. 1A). We hypothesized that *OCRL* is expressed in human tissues that are responsible for cataract and glaucoma development. To test this hypothesis, we examined human eyes previously enucleated for solitary choroidal melanoma that retained normal anterior and posterior segments. These sections were stained by H&E and immunohistochemistry was carried out with a previously characterized affinity-purified anti-OCRL antibody [35]. Indeed, OCRL was observed in punctate vesicles in the trabecular meshwork and lens epithelial cells; in addition, low level of protein expression was also detected in the Schlemm’s canal endothelial cells (Fig. 1B). In contrast, staining of eye sections using an anti-INPP5B antibody showed nearly undetectable signal in human trabecular meshwork as well the Schlemm’s canal endothelial cells.

The epithelial cells of the lens are a monolayer of epithelium with the basement membrane directed outwardly towards the anterior chamber. Similarly to the trabecular meshwork staining, OCRL is detected in a vesicular pattern but not INPP5B (Fig. 1C). The ciliary body epithelium, which is composed of outer pigmented and inner non-pigmented epithelium, was also examined for inositol phosphatase distribution. The non-pigmented epithelium (NPCE) is responsible for aqueous secretion and is the target for many anti-glaucoma medications. OCRL was detected in human ciliary body epithelium primarily in the outer pigmented epithelial cells; however, INPP5B protein was not detected (Fig. 1D). In the posterior segment of the eye, we found INPP5B immunoreactivity in the outer segment of the photoreceptors and retinal pigment epithelial cells (RPE); the pattern of localization corresponds to the outer photoreceptor segments (Fig. S1A-B in File S1). Similar immunohistochemical results for INPP5B and OCRL were obtained from rat eyes (data not shown).

*OCRL* and *INPP5B* are paralogous genes with overlapping enzymatic functions [19]. Although the loss of *OCRL* in humans results in severe multi-organ phenotypes, inactivation of *Ocrl* in mice does not phenocopy human disease. This may be due to redundancy of *Inpp5b,* which is supported by the early embryonic lethality of loss of both *Ocrl* and *Inpp5b*. Thus we compared the expression of *Ocrl* and *Inpp5b* in murine trabecular meshworks (MTM). The trabecular meshwork in murine eyes stained similarly for both OCRL and INPP5B (Fig. 1E). Using a purified antibody against INPP5B, we show the specificity by immunoblotting using lysates of hTERT-RPE1 knockdown of INPP5B (Fig. 1F). However, a comparison of their expression patterns in human trabecular meshwork (HTM) reveals that the protein level for OCRL was higher in HTM cells while INPP5B was relatively in MTM cells (Fig. 1G). Taken together, *OCRL* and *INPP5B* are differentially expressed in the ocular tissues involved in glaucoma and cataract development.

### INPP5B Localization to the Primary Cilia

**Figure 2:**
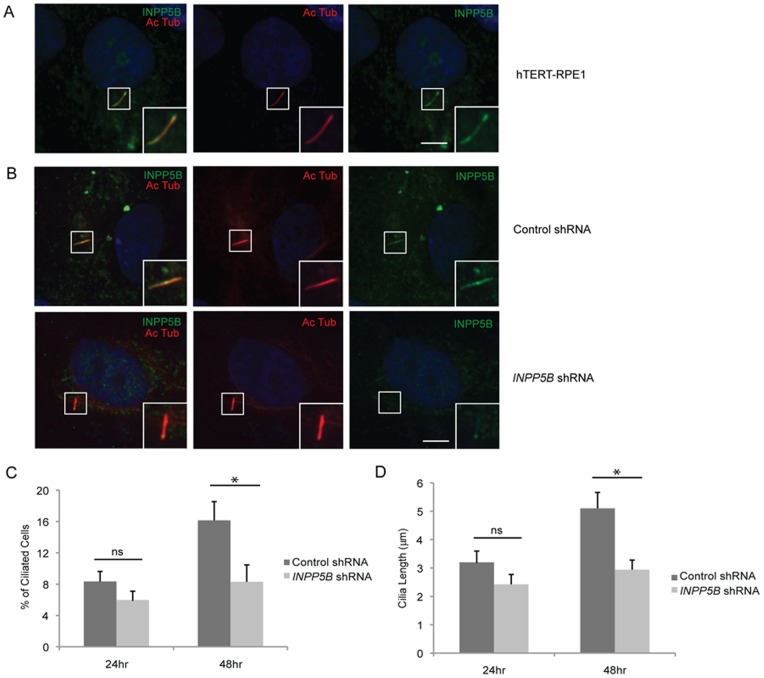
INPP5B localizes to primary cilia. (A) hTERT-RPE1 cells were starved for 48 hr, and analyzed by immunostaining with anti-INPP5B antibody and anti-acetylated alpha-tubulin antibody. Scale bar 5 micron. (B) Control and *INPP5B* shRNA hTERT-RPE1 cells were starved for 48 hr, and analyzed by immunostaining with anti-INPP5B antibody and anti-acetylated alpha-tubulin antibody. Scale bar 5 micron. (C-D) Control and *INPP5B* shRNA hTERT-RPE1 cells were starved for 24 hr or 48 hr, and analyzed by immunostaining with anti-acetylated alpha-tubulin antibody. Percent ciliated cells (C) and the length of cilia (D) were quantified. (n = the number of cilia n >100 cilia, three independent experiments, unpaired t-test, * p = 2.1E-08 in D, * p = 3.1 E-09 in E; ns, not statistically significant).

Because of the compensatory function of *Ocrl* and *Inpp5b* in mice, we hypothesized that INPP5B is also a ciliary protein. To address this possibility, we used hTERT-RPE1 cells, which is a well-described ciliated epithelial cell line [44]. Ciliogenesis was induced in hTERT-RPE1 cells by serum-starvation for 24 to 48 hr and the presence of INPP5B in the primary cilia was then examined with acetylated alpha-tubulin immunostaining. This showed that INPP5B localizes distinctly along the primary cilia (Fig. 2A). The immunostaining was absent in cells treated with shRNA *INPP5B* via a lentiviral vector, which resulted in an approximately 90% decrease in *INPP5B* expression as compared to controls (Fig. 2B–C). INPP5B was also detected with gamma-tubulin, a basal body marker (Fig. S2 in File S1).

Consequently, a role of INPP5B in cilia development was explored. *INPP5B* knockdown (KD) resulted in a significant decrease in the percentage of ciliated cells in comparison to control KD cells. Approximately 21% and 50% less ciliated cells were observed for *INPP5B* KD at 24 hour and 48 hour, respectively (8.2%±2% at 24 hour control KD vs 6.5%±1.5% in *INPP5B* KD cells, p = 0.04; 16%±2% at 48 hour control KD vs 8%±2% in *INPP5B* KD cells, p = 2.1E-08) (Fig. 2D). While decreased levels of INPP5B affected ciliation, we also assessed the cilia length in cells serum-starved at 24 and 48 hr. We found that the lengths of the cilia in *INPP5B* KD cells were significantly reduced as compared to control KD cells. After serum-starvation for 24 hr, hTERT-RPE1 control and *INPP5B* KD cells showed the cilia length to be 25% less than control cells (3.2±0.4 micron in control KD vs 2.4±0.3 micron in *INPP5B* KD cells, p = 0.1). About 45% less cells with *INPP5B* knockdown developed cilia when compared to controls cells after 48 hours of serum-starvation (5.1±0.6 micron in control KD vs 2.8±0.3 micron in *INPP5B* KD cells, p = 3.12E-09) (Fig. 2E). Therefore, INPP5B is important in development and regulation of primary cilia.

### Knockdown of Zebrafish INPP5B Results in Cilia Defects

**Figure 3:**
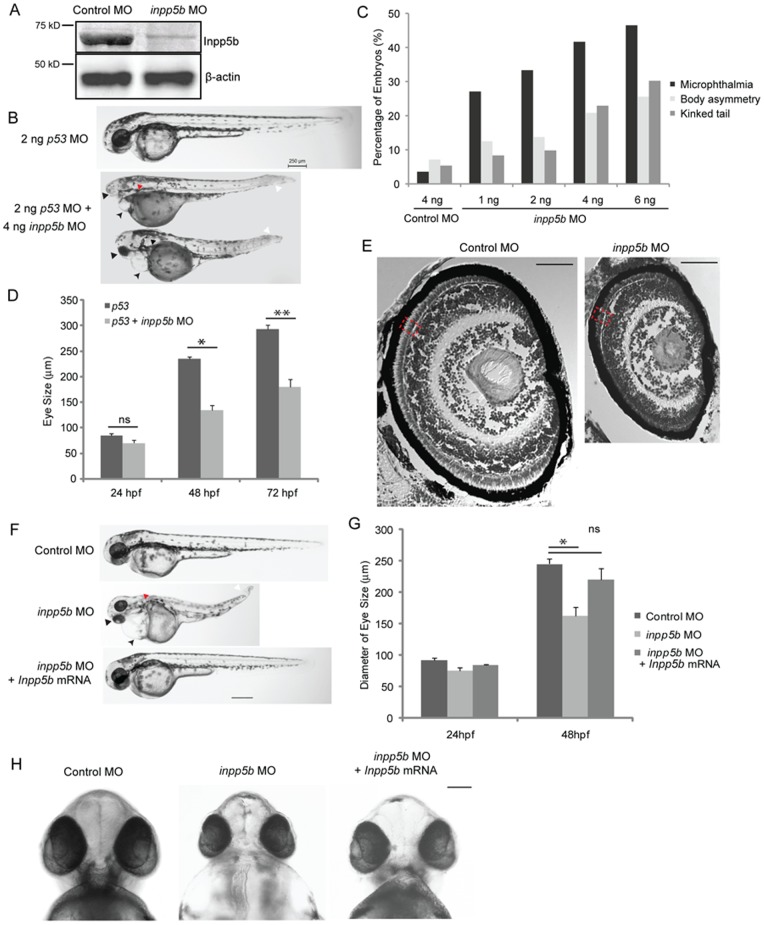
*Inpp5b* morpholino affects multiple organ development in zebrafish. (A) Immunoblot analysis of 40 microgram of total lysates of zebrafish embryo injected with control MO (4 ng) or *inpp5b* MO (4 ng) at 48 hpf with anti-INPP5B and anti-beta-actin antibodies. (B) Zebrafish embryos were injected with *p53* MO (2 ng) or *p53* MO (2 ng) and *inpp5b* MO (4 ng). Representative phenotypes of microphthalmia (black arrowhead), pericardial edema (small arrow), body axis asymmetry, kinked tail (white arrow), pronephric cyst formation (red arrow), and hypopigmentation were observed at 48 hpf. Scale bar 250 micron. (C) Dose-dependent effect of morpholinos in zebrafish. Control or *inpp5b* MO at indicated doses was injected into zebrafish embryos, and phenotypes of microphthalmia, kinked tail, and body asymmetry were quantified at 48 hpf (ANOVA, F = 92, p = 3.6E-10), kinked tail (ANOVA, F = 3.6, p = 0.08), and body asymmetry (ANOVA, F = 5.2, p = 0.04). (N = the number of injected embryos N >50). (D) Quantification of eye size of morphants at 24 hpf, 48 hpf and 72 hpf. The eye size was determined by the longest diameters in dorsal view. (N >40 embryos, three independent experiments, unpaired t-test, * p = 4.98E-07 ** p = 2.1E-10, ns, not statistically significant). (E) Cresyl violet staining of ocular sections of zebrafish larvae (5 dpf) injected with control MO (4 ng) or *inpp5b* MO (4 ng). Scale bar 30 micron. (F) Zebrafish embryos were injected with control MO (4 ng), *inpp5b* MO (4 ng) or *inpp5b* MO (4 ng) and *Inpp5b* WT mRNA (*Inpp5b* mRNA, 500 pg). Representative phenotypes of microphthalmia (arrowhead), pericardial edema (arrow), body axis asymmetry, kinked tail (white arrow), pronephric cyst formation (red arrow), and hypopigmentation were observed at 48 hpf. Scale bar 250 micron. (G) Quantification of eye size of zebrafish morphants at 24 hpf and 48 hpf. (N >40 embryos, three independent experiments, unpaired t-test, * p = 4.5E-05). (H) The ventral sides of embryos were injected with control MO (4 ng), *inpp5b* MO (4 ng) or *inpp5b* MO (4 ng) and *Inpp5b* WT mRNA (500 pg). Scale bar 100 micron.

To further determine the functional significance of *INPP5B* expression in cilia development, morpholino targeting *inpp5b* expression in zebrafish was employed. The degree of homolog between zebrafish and human INPP5B is approximately 61.0% identity and 76.0% similarity. For this purpose, an antisense oligonucleotide morpholino and a 5-base-pair mismatched control MO were designed and tested. In zebrafish, a single copy of *inpp5b* is present, which was conveniently targeted using antisense morpholinos. As shown in Figure 3A, *inpp5b* morpholino-injected embryos specifically decreased Inpp5b protein expression as compared to beta-actin. Expression of *inpp5b* was verified by immunoblot analysis to demonstrate the specificity of anti-INPP5B antibody. At the 48 hpf stage, *inpp5b* morphants exhibited dose-dependent microphthalmia, body axis asymmetry, and kinked tail; an approximately 70% of morphants exhibited microphthalmia, which were observed over multiple independent sets of experiments (Fig. 3B–C). To verify that the phenotypes are specific to *inpp5b* knockdown, *p53* MO were injected into zebrafish embryos. This showed that *p53* MO alone did not result in decreased eye size, generalized edema, or body axis asymmetry in zebrafish, but these phenotypes appeared in the embryos when co-injected with *inpp5b* MO and *p53* MO (Fig. 3B). These differences in eye size were statistically significant at 48 hpf between *p53* morphants and *p53* plus *inpp5b* morphants (236±3.5 micron vs 135±10 micron, p = 4.98E-07) (Fig. 3D). The significant difference in eye size was also found at 72 hpf between *p53* morphants and *p53* plus *inpp5b* morphants (293±9 micron vs 180±12 micron, p = 2.1E-10) (Fig. 3D).

Detailed analysis of these ocular phenotypes found the *inpp5b* morphants developed dystrophic retina and thinner RPE versus control (Fig. 3E). The phenotypes of edema, body asymmetry, and kinked tail in *inpp5b* morphants are characteristics of functional defects of cilia, and can be partly rescued by co-injection of murine *Inpp5b* (*Inpp5b*) WT mRNA with *inpp5b* MO (Fig. 3F–H). The eye size of morphants at 48 hpf co-injected with *Inpp5b* WT mRNA was 220±18 micron, compared with the morphants injected with *Inpp5b* MO (162±15 micron) and control MO (245±7.9 micron) (Fig. 3G).

**Figure 4:**
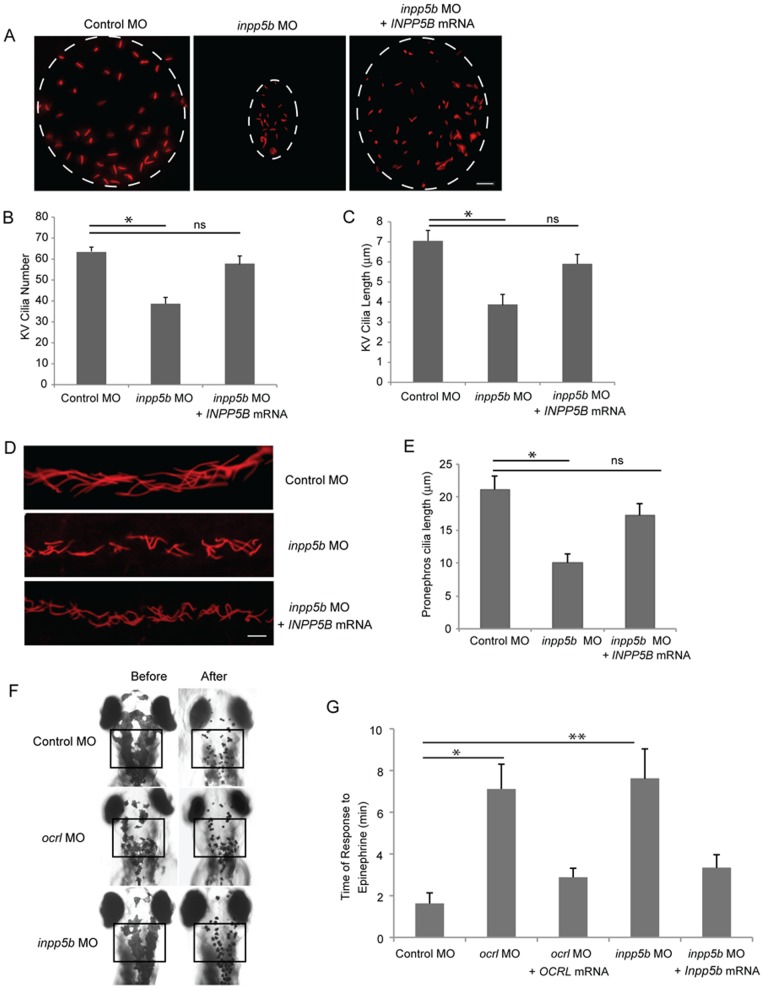
Defects of cilia formation in *Inpp5b* zebrafish morphants. (A) *INPP5B* WT mRNA rescue the loss of *inpp5b*. KV cilia of zebrafish embryos injected with control MO (4 ng), *inpp5b* MO (4 ng) or *inpp5b* MO (4 ng) and *INPP5B* WT mRNA (500 ng) at 6-somite stage were immunostained with acetylated α-tubulin (red), representative images are shown (dash line indicates border of KV). Scale bar 10 micron. (B–C) Quantification of number (B) and length (C) of KV cilia in zebrafish embryos injected with control MO (4 ng), *inpp5b* MO (4 ng) or *inpp5b* MO (4 ng) and *INPP5B* WT mRNA (500 ng). (N >20 embryos, three independent experiments, unpaired t-test, * p = 3.4E-03 in B and * p = 1.9E-23 in C). (D) *INPP5B* WT mRNA rescue of *inpp5b* pronephric cilia formation. Representative image of pronephric cilia of zebrafish embryos at 24 hpf stage, injected with control MO (4 ng), *inpp5b* MO (4 ng) or *inpp5b* MO (4 ng) and *INPP5B* WT mRNA (500 ng), immunostaining with acetylated α-tubulin (red). Scale bar 10 micron. (E) Pronephric cilia length of control and *inpp5b* MO. Pronephric cilia of zebrafish embryos injected with control MO (4 ng), *inpp5b* MO (4 ng) or *inpp5b* MO (4 ng) and *INPP5B* WT mRNA (500 ng) at 24 hpf stage were analyzed by immunostaining with acetylated alpha-tubulin and cilia length was measured. (N >200 cilia; three independent experiments, unpaired t-test, * p = 5.45E-18). (F) *Ocrl* and *Inpp5b* morphants showed slowed retrograde melanosome transport. Representative photos are shown for the melanosomes in *Ocrl* and *Inpp5b* morphants before and after treatment with epinephrine in 5 dpf embryos (box, region of pigment evaluation). (G) Quantification of the response time for epinephrine treatments in the control MO (2 ng), *ocrl* MO (2 ng), *ocrl* MO (2 ng) and *OCRL* WT mRNA (500 pg), *inpp5b* MO (2 ng), and *inpp5b* MO (2 ng) and *Inpp5b* WT mRNA (500 pg) embryos (N >30 embryos, three independent experiments, unpaired t-test, * p = 4.6E-50, ** p = 2.3E-43).

To evaluate whether Inpp5b is necessary for cilia development, we examined the Kupffer’s vesicle (KV) and pronephros duct of young zebrafish larvae. The KV is a ciliated, fluid-containing structure in the zebrafish, orthologous to the mouse embryonic node; it regulates left-right body axis and organ development through directional cilia rotation [45], [46]. KV was examined by immunohistochemistry with anti-acetylated alpha-tubulin to stain the cilia at the 6-somite stage (Fig. S3A in File S1). Compared with control MO-injected embryos, an approximate 50% reduction in the number of cilia in the KV of *inpp5b* morphants (64±2 vs 36±3 cilia, p = 3.41E-3) was detected (Fig. S3B in File S1). In addition, the average lengths of cilia within the KV were found to be shorter in the *inpp5b* morphants than controls embryos (4.3±0.5 micron vs 6.5±0.5 micron, p = 1.89E-23) (Fig. S3C in File S1). Cilia quantity (56±4) and lengths (6.2±0.5 micron) in the KV were rescued after co-injection with *Inpp5b* WT mRNA. Immunostaining of pronephric duct cilia with anti-acetylated alpha-tubulin at the 24 hpf stage was also carried out. Confocal images of individual cilium, with distinct base and ends (N >200 cilia), were outlined and the calculated length recorded. As compared to control MO-injected embryos, there was an approximately 50% shortening of cilia length in *inpp5b* morphants vs control embryos (9.5±1.8 micron vs 20±2.1 micron,p = 5.45E-18), and cilia length could be restored (17±1.9 micron) by co-injection with *Inpp5b* WT mRNA (Fig. S3D–E in File S1). Inpp5b is therefore concluded to exhibit an important role in cilia development and function during zebrafish embryogenesis. In zebrafish embryos co-injected with *Inpp5b* MO and human *INPP5B WT* mRNA, a reversal of ciliary phenotype was observed in KV cilia (Fig. 4A–C) and pronephric duct cilia (Fig. 4D–E). Compared with control MO-injected embryos, cilia number of KV return to 59±4 and average cilia length return to 5.7±0.6 micron. The pronephric cilia return to 17±2 micron.

Interestingly, we observed a decreased pigmentation in the *ocrl* and *inpp5b* morphants (Fig. 4F). These findings were consistent with our previous studies [42] with the *inpp5e* morphants suggesting that the *ocrl* and *inpp5b* morphant zebrafish may also have defects in melanosome transport. To measure melanosome transport, we determined the rates of epinephrine-induced melanosome retraction in the zebrafish larvae. When 5 dpf embryos were exposed to epinephrine, melanosomes contracted rapidly and the endpoint was marked by perinuclear accumulation of melanosomes [47]. Pigment accumulation of *ocrl* morphants (7.4±0.9 min) and *inpp5b* morphants (7.7±1.0 min) was 3 times longer than control morphants (1.8±0.4 min) (Fig. 4G). Importantly, the delayed retraction could be rescued by co-injecting either *OCRL* mRNA or *Inpp5b* mRNA (Fig. 4G).

### CAAX Domain of INPP5B Mediates Ciliary Localization

**Figure 5:**
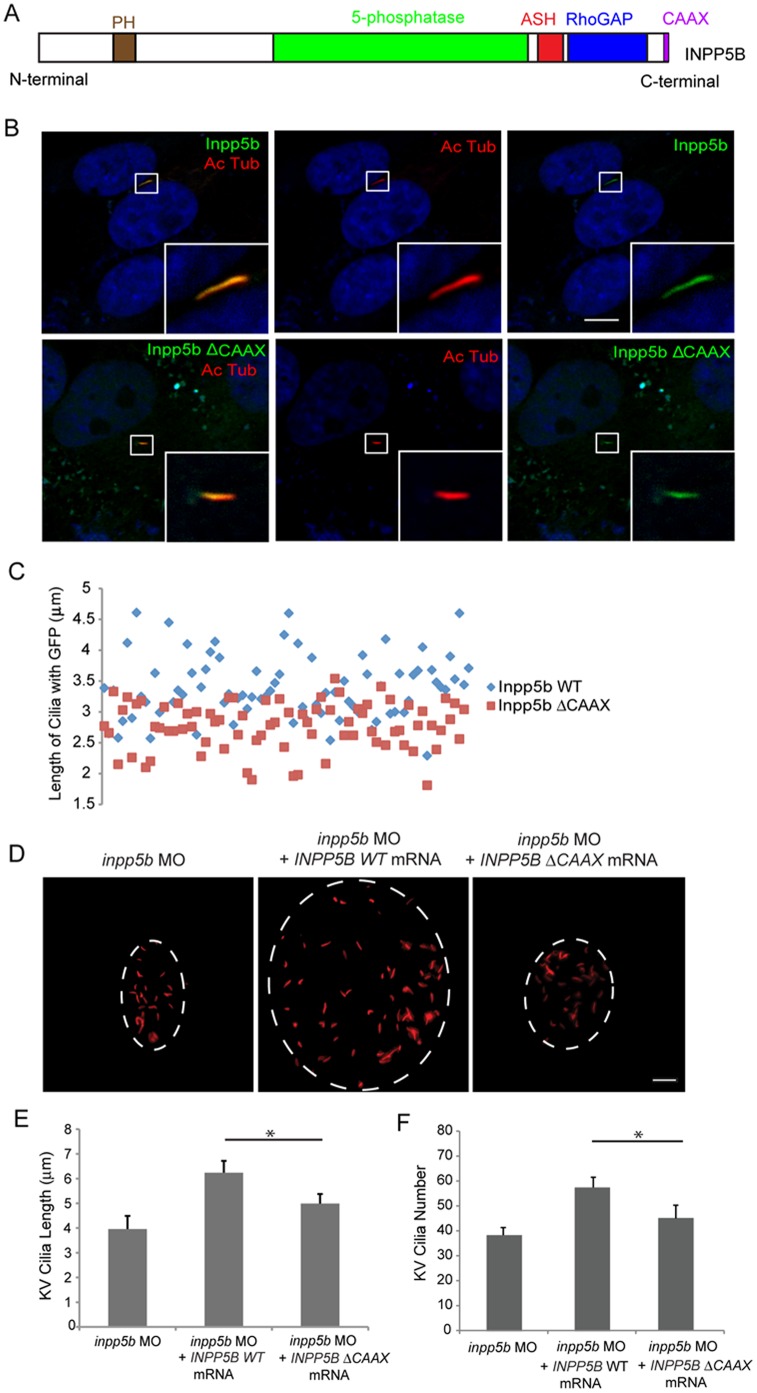
Effect of INPP5B CAAX mutant on cilia localization. (A) Domain structure of human INPP5B protein. (B) hTERT-RPE1 cells were transduced by *GFP-Inpp5b* or *GFP-Inpp5bΔCAAX* lentivirus, starved for 48 hr, and then analyzed by immunostaining with anti-acetylated alpha-tubulin antibody. Scale bar 10 micron. (C) Lengths of primary cilia in *Inpp5b* and *Inpp5b*-*delta*-CAAX cells. hTERT-RPE1 cells were transduced with either *GFP-Inpp5b* or *GFP-Inpp5b*-*delta*-*CAAX* lentivirus, serum starved for 48 hr, and stained with anti-acetylated alpha-tubulin antibody. Scatter plot showing distribution pattern of ciliary length (3.4±0.4 micron in *INPP5B* and 2.8±0.3 micron in *Inpp5bΔCAAX* cells, unpaired t-test, p = 1.36E-06, n >160 cilia, three independent experiments). (D) *INPP5BΔCAAX* mRNA failed to rescue the loss of *inpp5b*. KV cilia of zebrafish embryos injected with *inpp5b* MO (4 ng), *inpp5b* MO (4 ng) with *INPP5B* WT mRNA (500 ng) and *inpp5b* MO (4 ng) with *INPP5B ΔCAAX* mRNA (500 ng) at 6-somite stage were immunostained with acetylated α-tubulin (red), representative images are shown (dash line indicates border of KV). Scale bar 10 micron. (E–F) Quantification of length (E) and number (F) of KV cilia in zebrafish embryos injected with *inpp5b* MO (4 ng), *inpp5b* MO (4 ng) with *INPP5B* WT mRNA (500 ng) and *inpp5b* MO (4 ng) with *INPP5B ΔCAAX* mRNA (500 ng). (N >20 embryos, three independent experiments, unpaired t-test, * p = 3.9E-26 in E and * p = 9E-06 in F).

Inositol phosphatase *INPP5E* contains a prenylation CAAX motif, which was found to play a role in MORM syndrome (a ciliopathy) [27]. A premature stop codon in *INPP5E* affects ciliary localization but not its enzymatic activity. For this purpose, a CAAX deletion mutant of Inpp5b was assayed for its ability to be recruited to the cilia (Fig. 5A). In hTERT-RPE1 cells transduced with GFP-tagged lentivirus for either *Inpp5b* WT or *Inpp5b*-*delta-CAAX*, the localization of this protein by immunofluorescence was visualized (Fig. 5B). The expression of *Inpp5b* or Inpp5b-*delta-CAAX* was verified by immunoblot analysis (data not shown). In 48 hr serum-starved hTERT-RPE1 cells expressing either *Inpp5b* or *Inpp5b*-*delta-CAAX*, the loss of the CAAX motif significantly abolished the ciliary recruitment of INPP5B. In hTERT-RPE1 cells transduced with either *Inpp5b* or *Inpp5b*-*delta-CAAX* lentivirus, the length of GFP-positive cilia was assessed and a significant difference was noted (3.4±0.4 micron vs 2.8±0.3 micron, p = 1.36E-06) (Fig. 5 C).

The ability of *Inpp5b*-delta-CAAX and *INPP5B*-*delta*-*CAAX* mutant to rescue the loss of INPP5B was investigated. Since those cilia-associated phenotypes in *Inpp5b* morphants can be partly rescued by co-injection *Inpp5b* WT mRNA with *inpp5b* MO, an *Inpp5b*-*delta*-CAAX mutant of mRNA was co-injected with 4 ng *inpp5b* MO (Fig. S3F-G in File S1). In *Inpp5b* morphants co-injected with *Inpp5b* WT mRNA, the KV cilia length was 6.5±0.5 micron; however, the KV cilia in morphants co-injected with *Inpp5b*-*delta*-*CAAX* mRNA showed shortened cilia (4.7±0.4 micron, p = 4.90E-07) (Fig. S3G in File S1). Compared with morphants co-injected with *INPP5B* WT mRNA, the KV cilia in embryos co-injected with *INPP5B*-*delta*-*CAAX* mRNA was 44±5 (p = 3.90E-26, Fig. 5E) and 4.4±0.5 micron in length (p = 9E-06, Fig. 5F). Therefore, the CAAX domain is important for INPP5B in the formation of primary cilia.

### Compensatory Role of OCRL and INPP5B in Ciliogenesis in Zebrafish

**Figure 6:**
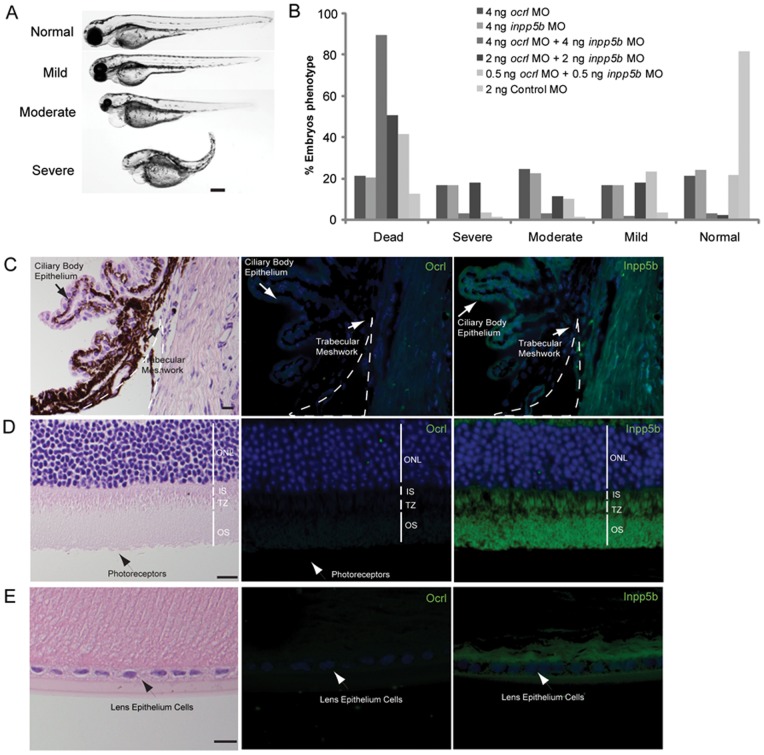
INPP5B and OCRL act synergistically in eye development. (A) Representative phenotypes of morphants co-injected with *ocrl* and *inpp5b* MO. Scale bar 250 micron. (B) Dose-dependent effect of two morpholinos in zebrafish. Control *ocrl* and *inpp5b* MO at indicated doses were injected into zebrafish embryos, and different grades of phenotypes were quantified at 48 hpf (N >250 embryos per group). (C) Transgenic *mOcrl^−/−^:mInpp5b^−/−^:hINPP5B* ^+/+^ mouse eyes were sectioned and stained by H&E, anti-OCRL antibody (green) or anti-INPP5B antibody (green), and DAPI (blue). Staining of trabecular meshwork cells in vesicular pattern (arrow), dash line indicates border of cornea and iris. Scale bar 10 micron. (D) Photoreceptor cells (arrow) from the transgenic mouse eye section stained with H&E, anti-OCRL antibody (green) or anti-INPP5B antibody (green), and DAPI (blue). Scale bar 10 micron. (E) Lens epithelial cells (arrow) stained with H&E, anti-OCRL antibody (green) or anti-INPP5B antibody (green), and DAPI (blue). Scale bar 10 micron.

The similar function and structures *OCRL* and *INPP5B* suggests that the knockdown of both 5-phosphatases may result in greater ciliary defects than the loss of either enzyme alone. To make such comparisons, a scale of phenotypes observed in zebrafish morphants, which include anophthalmia, microphthalmia, hydrocephalus, left-right axis asymmetry, generalized edema, and pigmentation defects, was established. By this scale, the normal fish was indistinguishable from the wild type fish. This scale consists of mild, with one or two of the phenotypes; moderate, with greater than two of the cilia-related phenotypes; or severe, if four or more abnormalities were present (Fig. 6A). In zebrafish treated with either 4 ng of *ocrl* or *inpp5b* MO, approximately 20% of zebrafish developed severe disease whereas when both MO are given at 4 ng, nearly 90% of zebrafish were dead within 54 hours (Fig. 6B). The combination of 2 ng *ocrl* and *inpp5b* MO resulted in equivalent number of severe phenotype as the 4 ng groups; whereas 0.5 ng of *ocrl* and *inpp5b* MO also resulted in equal numbers of mild phenotypes in morphants (Fig. 6B). Therefore, we conclude Ocrl and Inpp5b play synergistic roles in zebrafish; the loss of both genes results in a greater degree of phenotype than either gene alone.

The transgenic murine model of Lowe syndrome was also examined. Mice with *Ocrl* ^−/−^:*Inpp5b* ^−/−^:*INPP5B* ^+/+^ genotype are previously characterized to show renal phenotypes resembling human disease and that introduction of INPP5B rescues the lethality of the double *Ocrl/Inpp5b* knockout. In these mice, the loss of *Ocrl* was compensated by *Inpp5b* expression as determined by immunohistochemistry showing normal structures of the trabecular meshwork, lens epithelial cells, and retinal outer segment (Fig. 6C–E). Expression of *Inpp5b* was detected in these tissues while *Ocrl* was not observed. Thus, human INPP5B likely compensates for the loss of OCRL in ocular tissue in this murine model.

**Figure 7:**
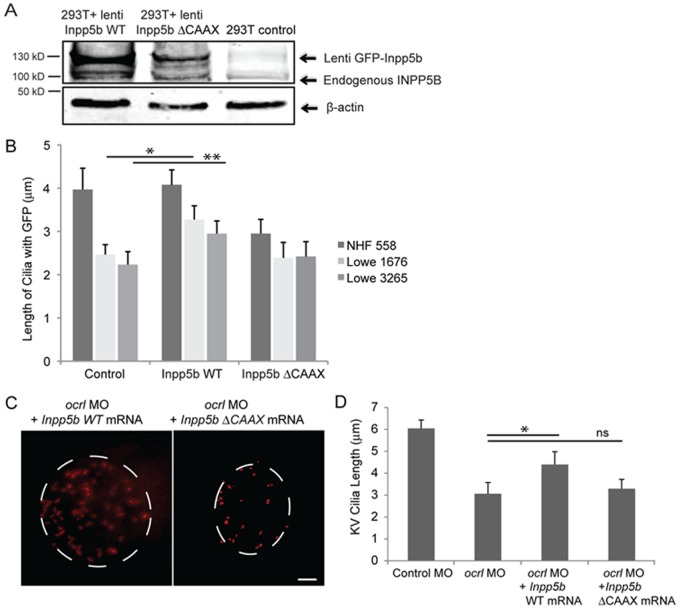
INPP5B can partly rescue the defect of OCRL in primary cilia development. (A) INPP5B protein levels in HEK293T cells. *GFP-Inpp5b* lentivirus was generated in HEK293T cells; both GFP-Inpp5b and endogenous INPP5B were immunoblotted in 40 microgram lysates; beta-actin levels are shown. (B) NHF 558, Lowe 1676, and Lowe 3265 fibroblasts were transduced with control, *Inpp5b* or *Inpp5b ΔCAAX* lentivirus, serum-starved for 48-hours, and immunostained with acetylated alpha -tubulin. Quantification of cilia length is shown (n >100 cilia, three independent experiments, unpaired t-test, * p = 0.004, ** p = 0.006). (C) *Inpp5b* mRNA partly rescued the loss of *Ocrl*. KV cilia of zebrafish embryos injected with *ocrl* MO (4 ng) with *Inpp5b* WT mRNA (500 ng) and *ocrl* MO (4 ng) with *Inpp5bΔCAAX* mRNA (500 ng) at 6-somite stage were immunostained with acetylated α-tubulin (red), representative images are shown (dash line indicates border of KV). Scale bar 10 micron. (D) Quantification of length of KV cilia in zebrafish embryos injected with *ocrl* MO (4 ng) with *Inpp5b* WT mRNA (500 ng) and *ocrl* MO (4 ng) with *Inpp5bΔCAAX* mRNA (500 ng). (N >20 embryos, three independent experiments, unpaired t-test, * p = 2.2E-04).

To confirm compensatory roles between OCRL and INPP5B, we examined whether INPP5B could restore ciliary length in Lowe patients fibroblasts previously characterized [35], [36], [37]. Normal human fibroblasts, Lowe 1676 and Lowe 3265 fibroblasts were transduced with *GFP-Inpp5b* or *GFP-Inpp5b*-*delta*-*CAAX* lentivirus, and cilia formation was induced by serum starvation for 48 hr. Acetylated alpha-tubulin immunostaining was performed to determine ciliary length (Fig. 7A). In Lowe 1676 patient fibroblasts, cilia lengths were noted to be longer in *Inpp5b* transduced cells than those treated with control lentivirus (3.3±0.3 micron vs 2.4±0.2 micron, p = 0.004). Similarly, in Lowe 3265 patient fibroblasts, cilia lengths were also noted to be longer in *Inpp5b* transduced cells than the control cells (2.2±0.2 micron vs 3±0.3 micron, p = 0.006) (Fig. 7B). However, *Inpp5b*-*delta*-*CAAX* domain did not restore the ciliary length (2.4±0.4 micron in Lowe 1676 and 2.5±0.4 micron in Lowe 3265) (Fig. 7B).

Additionally, zebrafish *inpp5b* was assessed for its ability to restore the loss of cilia formation in *ocrl* morphant zebrafish. The KV cilia in morphants co-injected with *ocrl* MO and *Inpp5b* WT mRNA were noted to be longer than those injected with *ocrl* MO (4.3±0.4 micron vs 3.0±0.3 micron, p = 2.19E-04) (Fig. 7C). Co-injection with *Inpp5b*-*delta*-*CAAX* mRNA did not restore the cilia length in the *ocrl* morphants (3.2±0.2 micron vs 3.0±0.3 micron, p = 0.07) (Fig. 7D). Therefore, we conclude that INPP5B can rescue the role of OCRL in development and regulation of primary cilia.

## Discussion

The ciliary membrane is comprised of a wide range of proteins and lipids that are highly regulated. Previously inositol phosphatases OCRL and INPP5E have been found to be implicated in Lowe syndrome and Joubert syndrome, respectively. Here, the paralog of *OCRL*, *INPP5B* is also found to be a ciliary protein with compensatory roles in ciliogenesis in RPE cells and in zebrafish. Evidence of differential expression of *OCRL* and *INPP5B* in human and mice is also presented that potentially explains the mild phenotype of Lowe syndrome murine models.

INPP5B has been previously suggested to play a role in ciliogenesis [36]. This study shows that inositol 5-phosphatase localizes within the cilia and the localization is dependent on the CAAX prenylation domain. *INPP5E* deletion of CAAX domain has been reported in MORM syndrome, another ciliopathy with clinical similarities to Bardet-Beidel syndromes [27]. Recently Humbert *et al*. has reported a ciliary targeting sequence (FDRELYL) that precedes the CAAX domain in INPP5E [34]; however, a homology search did not yield a similar sequence in INPP5B. In this study the CAAX domain of *INPP5B* is demonstrated to be important in its localization and its ability to regulate ciliary length *in vitro* and *in vivo*. This suggests that the enzymes controlling prenylation are key regulator for INPP5B and INPP5E ciliary function. Based on this observation, the type I 5-phosphatase, which also contains a CAAX domain, is also predicted to localize to the cilia.

In murine knockout studies, loss of *Ocrl* failed to show phenotypes of cataract, glaucoma, renal failure, or brain abnormalities. Mice with *Inpp5b* loss resulted in spermatogenesis defect but *Ocrl^−/−^*:*Inpp5b^−/−^* resulted in embryonic lethality. The ocular distribution of these two 5-phosphatases observed here may explain the lack of ocular phenotype in the mice. In murine eyes, both *Ocrl* and *Inpp5b* are expressed in relatively equal levels; however, by both immunofluorescence and immunoblot detection, *Ocrl* is the predominant 5-phosphatase in the trabecular meshwork and lens epithelium. In *Ocrl^−/−^* mice, Inpp5b likely compensates for the loss of Ocrl and consistently no obvious ocular phenotypes were observed. Further, in the zebrafish, Inpp5b is found to regulate ciliogenesis in both the KV and pronephros in early embryogenesis. In fact, the loss of *Inpp5b* results in a more severe phenotype of anophthalmia, which is not present in the *ocrl*-MO injected fish, and suggests *inpp5b* is required in cilia development in the zebrafish.

Functionally, *ocrl* and *inpp5b* act synergistically in zebrafish cilia development. The loss of both 5-phosphatases results in greater percentage of lethality and severe phenotypes of the morphant fish than the loss of either of the enzymes. Interestingly, although *inpp5e* is unperturbed in these morphants, it is unable to prevent the development of these phenotypes. Our studies of human retina and RPE show that INPP5B is expressed in the outer segment of the photoreceptors and the RPE. Although absent from trabecular meshwork and lens epithelium, INPP5B in the retina may explain the lack of retinitis pigmentosa (RP) phenotype in Lowe syndrome. Clinically RP is not observed in Lowe syndrome however the vision of patients is abnormally poor, even with cataract surgery and glaucoma treatment. In fact, in certain Lowe syndrome patients we observe thinning of the outer retina, suggesting mild retinal disease may not be previously reported (Luo *et al*. manuscript in preparation). The compensation of *INPP5B* for *OCRL* in the retina could account for the lack of pigmentary changes seen in RP. It is possible that the loss of both *OCRL* and *INPP5B* in humans may result in anophthalmia as in the zebrafish model. In addition, mutations of *INPP5B* may be present in patients with a subgroup of population of RP patients.

The ocular distribution of OCRL shows the expected vesicular pattern in the trabecular meshwork, which contributes significantly to the outflow of aqueous humor. When the trabecular meshwork is dysfunctional, the resistance to aqueous humor outflow increases with a concomitant increase in intraocular pressure. Increased intraocular pressure is the leading risk factor for glaucoma and a leading causative factor for the degenerative optic neuropathy of glaucoma. The glaucoma phenotype present in Lowe syndrome may be a dysregulation of the cilia in the aqueous outflow track and may also represent a *forme fruste* for other forms of glaucoma [4]. The lenticular changes found in Lowe syndrome–small discoid cataracts–likely representation of failure of primary ciliogenesis of the lens epithelial cells. The immunohistochemistry of the human lens epithelial cells shows vesicular pattern that is expected of endosomes. The defective cilia may result in polarity dysregulation, which leads to a discoid cataract. Based on our results, we propose that the diverse phenotype present in Lowe syndrome is due to the dysregulation of the primary cilia, leading to polarity defects in a number of cell types and thus explains the spectrum and type of organ involvement in this genetic disorder.

Important questions remain in the mechanism of inositol 5-phosphatase regulation of cilia. Although CAAX domain is important in the ciliary localization of INPP5B and INPP5E, the critical enzyme and underlying mechanism for this targeting is not known. The role of phosphoinositides within the cilia is also not clear. Although PI(4,5)P_2_ and PI(3,4,5)P_3_ are likely the critical lipids within the cilia, the differential regulation of these lipid messengers in lens development, glaucoma pathogenesis, and retinal development is not clear. Future therapeutic targets to upregulate *INPP5B* in the trabecular meshwork and the lens epithelium may help ameliorate or even reverse the clinical development of ocular phenotypes in Lowe syndrome.

## Supporting Information

File S1 **Includes Figures S1, S2 and S3. Figure S1 Distribution of INPP5B in human retina and RPE.** (A) Retina pigmented epithelial cells (arrow) from human eye section stained with H&E or anti-INPP5B antibody (green), and DAPI (blue). Scale bar 10 micron. (B) Photoreceptor cells (arrow) from human eye section stained with H&E or anti-INPP5B antibody (green), and DAPI (blue). Scale bar 10 micron. **Figure S2 Subcellular distribution of INPP5B in cilia.** hTERT-RPE1 cells serum starved for 48 hr and immunostained for acetylated alpha-tubulin, gamma-tubulin anti-INPP5B and DAPI. Representative image is shown (Red, alpha-tubulin, gamma-tubulin; green, INPP5B; blue, DAPI). **Figure S3 Restoration of cilia defects in** ***Inpp5b*** **zebrafish morphants by mouse** ***Inpp5b*** **mRNA.** (A) *Inpp5b* WT mRNA rescued the loss of *inpp5b*. KV cilia of zebrafish embryos injected with control MO (4 ng), *inpp5b* MO (4 ng) or *inpp5b* MO (4 ng) and *Inpp5b* WT mRNA (500 ng) at 6-somite stage were immunostained with acetylated alpha-tubulin (red), representative images are shown (dash line indicates border of KV). Scale bar 10 micron. (B-C) Quantification of number (B) and length (C) of KV cilia in zebrafish embryos injected with control MO (4 ng), *inpp5b* MO (4 ng) or *inpp5b* MO (4 ng) and *Inpp5b* WT mRNA (500 ng). (D) *Inpp5b* WT mRNA rescue of *inpp5b* pronephric cilia formation. Representative image of pronephric cilia of zebrafish embryos at 24 hpf stage, injected with control MO (4 ng), *inpp5b* MO (4 ng) or *inpp5b* MO (4 ng) and *Inpp5b* WT mRNA (500 ng), immunostaining with acetylated α-tubulin (red). Scale bar 10 micron. (E) Pronephric cilia length of control and *inpp5b* MO. Pronephric cilia of zebrafish embryos injected with control MO (4 ng), *inpp5b* MO (4 ng) or *inpp5b* MO (4 ng) and *Inpp5b* WT mRNA (500 ng) at 24 hpf stage were analyzed by immunostaining with acetylated alpha-tubulin and cilia length was measured. (F) *Inpp5b-delta-CAAX* mRNA failed to rescue the loss of *inpp5b*. KV cilia of zebrafish embryos injected with *inpp5b* MO (4 ng), *inpp5b* MO (4 ng) with *Inpp5b* WT mRNA (500 ng) and *inpp5b* MO (4 ng) with *Inpp5b-delta-CAAX* mRNA (500 ng) at 6-somite stage were immunostained with acetylated alpha-tubulin (red), representative images are shown (dash line indicates border of KV). Scale bar 10 micron. (G) Quantification of length of KV cilia in zebrafish embryos injected with *inpp5b* MO (4 ng), *inpp5b* MO (4 ng) with *Inpp5b* WT mRNA (500 ng) and *inpp5b* MO (4 ng) with *Inpp5b-delta-CAAX* mRNA (500 ng) (6.5+0.5 micron in morphants of *inpp5b* MO with *Inpp5b* mRNA compared to 4.7+0.4 micron in morphants of *inpp5b* MO with *Inpp5b-delta-CAAX* mRNA, unpaired t-test, * p = 4.9E-07, N >20 embryos, three independent experiments).
(DOCX)
