# Supplementary figures and images for: Compensatory Role of Inositol 5-Phosphatase INPP5B to OCRL in Primary Cilia Formation in Oculocerebrorenal Syndrome of Lowe
Source: PLoS One. 2013 Jun 21;8(6):e66727. doi: 10.1371/journal.pone.0066727 (PMC3689662; doi:10.1371/journal.pone.0066727)

Supporting Information


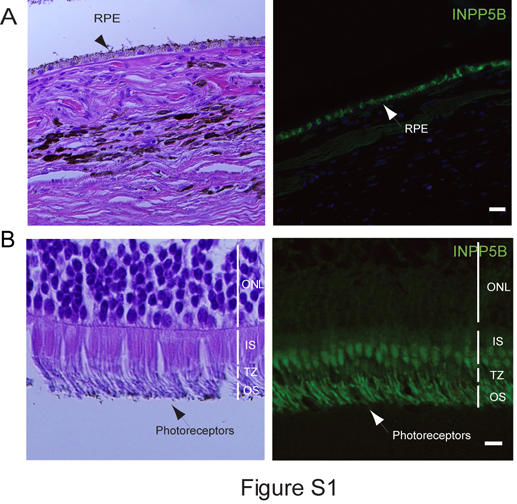


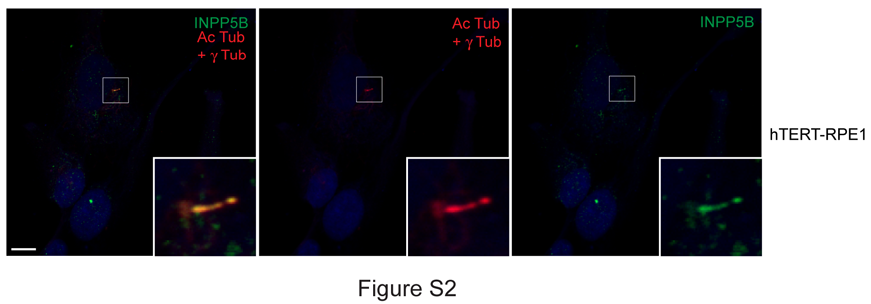


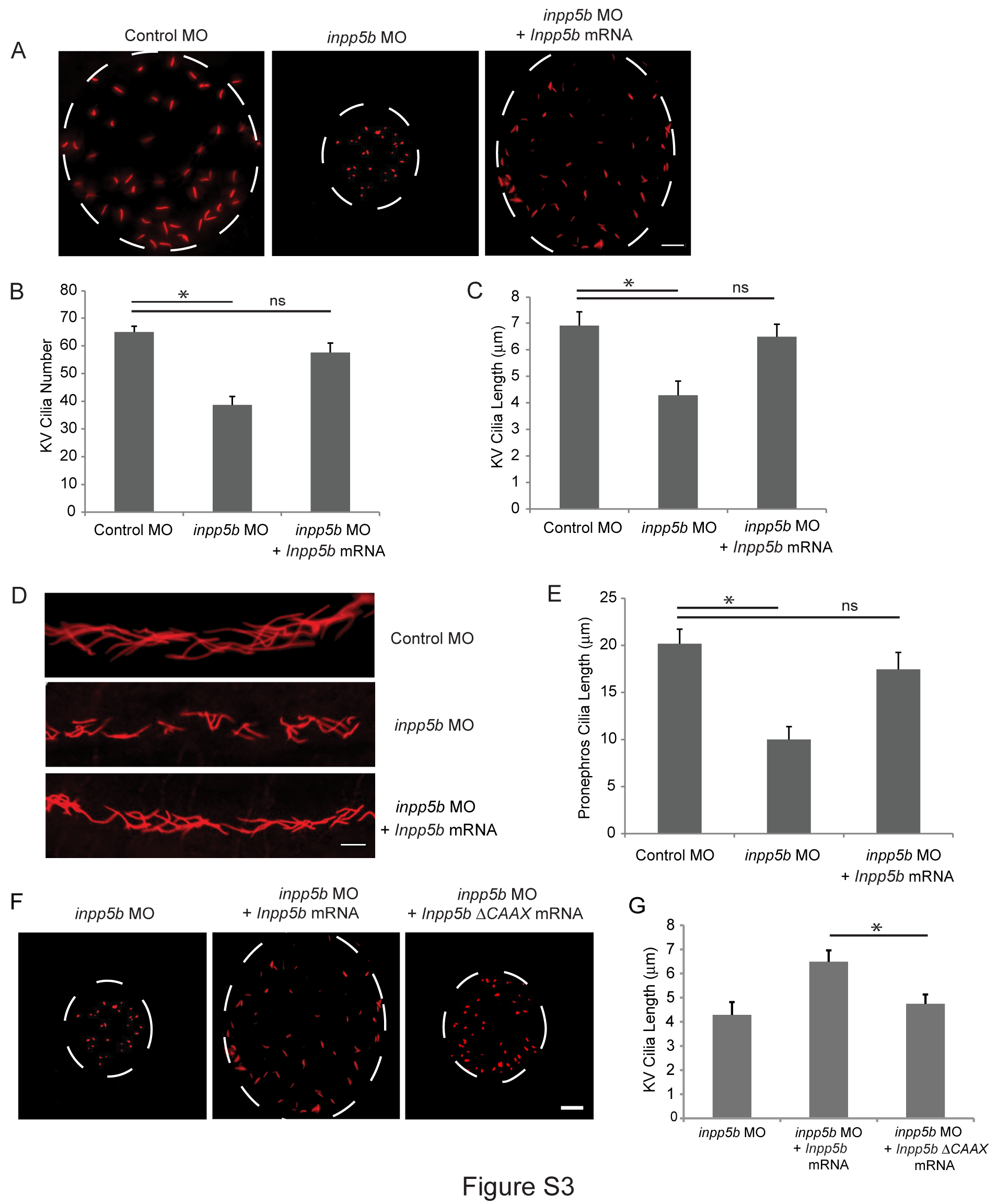

Supplement: File S1 — Includes Figures S1, S2 and S3. Figure S1 Distribution of INPP5B in human retina and RPE. (A) Retina pigmented epithelial cells (arrow) from human eye section stained with H&E or anti-INPP5B antibody (green), and DAPI (blue). Scale bar 10 micron. (B) Photoreceptor cells (arrow) from human eye section stained with H&E or anti-INPP5B antibody (green), and DAPI (blue). Scale bar 10 micron. Figure S2 Subcellular distribution of INPP5B in cilia. hTERT-RPE1 cells serum starved for 48 hr and immunostained for acetylated alpha-tubulin, gamma-tubulin anti-INPP5B and DAPI. Representative image is shown (Red, alpha-tubulin, gamma-tubulin; green, INPP5B; blue, DAPI). Figure S3 Restoration of cilia defects in Inpp5b zebrafish morphants by mouse Inpp5b mRNA. (A) Inpp5b WT mRNA rescued the loss of inpp5b. KV cilia of zebrafish embryos injected with control MO (4 ng), inpp5b MO (4 ng) or inpp5b MO (4 ng) and Inpp5b WT mRNA (500 ng) at 6-somite stage were immunostained with acetylated alpha-tubulin (red), representative images are shown (dash line indicates border of KV). Scale bar 10 micron. (B-C) Quantification of number (B) and length (C) of KV cilia in zebrafish embryos injected with control MO (4 ng), inpp5b MO (4 ng) or inpp5b MO (4 ng) and Inpp5b WT mRNA (500 ng). (D) Inpp5b WT mRNA rescue of inpp5b pronephric cilia formation. Representative image of pronephric cilia of zebrafish embryos at 24 hpf stage, injected with control MO (4 ng), inpp5b MO (4 ng) or inpp5b MO (4 ng) and Inpp5b WT mRNA (500 ng), immunostaining with acetylated α-tubulin (red). Scale bar 10 micron. (E) Pronephric cilia length of control and inpp5b MO. Pronephric cilia of zebrafish embryos injected with control MO (4 ng), inpp5b MO (4 ng) or inpp5b MO (4 ng) and Inpp5b WT mRNA (500 ng) at 24 hpf stage were analyzed by immunostaining with acetylated alpha-tubulin and cilia length was measured. (F) Inpp5b-delta-CAAX mRNA failed to rescue the loss of inpp5b. KV cilia of zebrafish embryos injected with inpp5b [file pone.0066727.s001.docx]
